# Editorial: Natural medicines for metabolic diseases – computational and pharmacological approaches, Volume II

**DOI:** 10.3389/fphar.2026.1799670

**Published:** 2026-02-27

**Authors:** Antony Stalin, Christudas Sunil, Abd El-Latif Hesham, Savarimuthu Ignacimuthu, Quan Zou, Yansu Wang

**Affiliations:** 1 Institute of Fundamental and Frontier Sciences, University of Electronic Science and Technology of China, Chengdu, China; 2 Cellular and Molecular Biology, UT Tyler School of Medicine, Tyler, TX, United States; 3 Genetics Department, Faculty of Agriculture, Beni-Suef University, Beni-Suef, Egypt; 4 Xavier Research Foundation, St Xavier’s College, Palayamkottai, India

**Keywords:** artificial intelligence, bioinformatics (computational biopharmaceutics and modeling), metabolic diseases, natural compounds, network pharmacology, pharmaceutical chemistry

Metabolic conditions, including type 2 diabetes mellitus (T2DM), obesity, dyslipidemia, and metabolic dysfunction associated with steatotic liver disease, together with their cardiovascular and renal complications, continue to rise in prevalence and impose a substantial economic burden worldwide. Current estimates indicate that more than 500 million individuals are affected by diabetes globally and is projected to increase markedly by 2045 (Sun et al., 2022). The multi-organ and multi-pathway dysregulation characteristic of these disorders leads to clinical heterogeneity, making it difficult to select treatment targets and resulting in mixed responses.

Small molecules extracted from natural products and traditional medicines remain an important source of therapeutics, particularly for pathway-level modulation and rational polypharmacology (Newman and Cragg, 2020; Atanasov et al., 2021). In parallel, systems pharmacology, multi-omics technologies, and data-driven artificial intelligence (AI) methods are transforming the field by bridging phytochemicals, molecular targets, and disease phenotypes, enabling exploration of large chemical spaces and generation of experimentally testable mechanistic hypotheses (Hopkins, 2008; Vamathevan et al., 2019). Together, these advances provide a more tractable and mechanism-based framework for natural product research, from dataset construction and model development to candidate prioritization and biological validation.

The Research Topic Natural Medicines for Metabolic Diseases: Computational and Pharmacological Approaches brings together contributions that integrate computational and experimental strategies to accelerate translation. It continues the editorial vision introduced in Volume I of this series (Stalin et al., 2024). Volume II comprises six papers: three original research articles and three review or meta-analytic studies, covering diabetic complications, target discovery, and methodology development.

Rao et al. present a multi-omics investigation of the anti-colorectal cancer activity of formononetin, a prominent bioactive constituent of *Hedysari Radix*. By integrating proteomics and metabolomics, the study demonstrates that formononetin modulates signaling pathways involved in cell proliferation, apoptosis, inflammation, and metabolic reprogramming in colorectal cancer. The layered omics strategy enhances interpretability by producing convergent evidence across independent molecular datasets and supports network-level, testable mechanistic predictions. Beyond its specific findings, this work illustrates how multi-omics approaches can increase mechanistic confidence and improve target prioritization in complex disease contexts.

Diabetic kidney disease (DKD) represents a major contributor to morbidity and mortality among patients with diabetes, and lipid accumulation associated with impaired lipophagy is increasingly recognized as a key driver of renal lipotoxicity and disease progression. Gao et al. review evidence demonstrating that plant-derived bioactive compounds can modulate the autophagy–lysosome axis and restore lipid homeostasis through regulators such as AMPK, mTOR, PPARs, and TFEB. The review offers a consistent mechanistic framework for experimental design and disease treatment, highlighting the importance of lipophagy regulation in renal protection and metabolic remodeling, and identifying opportunities for multi-pathway intervention with natural products.

Organ-specific vulnerability to metabolic stress is also evident in diabetic retinopathy, where mitochondrial dysfunction and defective mitophagy play critical pathogenic roles. Zhang et al. identify luteolin as a regulator of the SQSTM1/BNIP3L axis and demonstrate that enhancing mitophagic flux may help reduce oxidative stress and retinal cellular damage. This review of a specific mitochondrial quality-control axis makes mitophagy-targeted intervention more plausible and supports further consideration of naturally occurring flavonoids as potential agents for microvascular complications.

Advances in computational target discovery are exemplified by Liao et al. who introduce a model based on graph autoencoders (GAEs) and non-negative matrix factorization (NMF) to characterize metabolite–disease relationships and uncover latent pathological structures in metabolic disorders. The model demonstrates strong predictive performance and identifies metabolite-centered pathways relevant to diabetes and related conditions by learning discriminative representations from known associations and embedding them within network architectures. The value of this work lies not only in its predictive accuracy but also in its ability to generate hypotheses by identifying mechanisms involving metabolites that may be sensitive to manipulation by natural medicines.

Wang et al. describe a multimodal computational pipeline for screening natural compounds that co-target adenosine receptor subtypes A1 and A2A, G protein–coupled receptors involved in glucose and lipid metabolism, inflammation, and cellular stress responses. Their workflow integrates hERG-based toxicity filtering, QSAR-based activity prediction, and deep generative modeling using stacked long short-term memory (LSTM) networks. Balance among selectivity, drug-likeness, and synthetic accessibility is achieved through reinforcement learning and Pareto optimization, prioritizing candidates with favorable predicted pharmacokinetic properties. This research demonstrates the use of AI-based workflows to support multi-objective decision-making for metabolic goals, where efficacy and safety must be co-optimized.

Cautious evidence synthesis also benefits clinical translation. Lin et al. conducted a systematic review and meta-analysis of Qingre Lishi decoction (QRLSD) for T2DM, summarizing data from 18 randomized controlled trials. The analysis reports improvements in fasting and postprandial glucose, HbA1c, lipid profiles, and indices of insulin resistance, without an apparent increase in adverse events. However, substantial heterogeneity among studies and variability in formulations limit the strength of causal inference. The authors appropriately call for large, multicenter trials using standardized preparations and extended follow-up to assess generalizability and long-term benefit.

In Volume II, several priorities emerge for the next stage. First, improved data infrastructure is essential: standardized chemical identifiers, harmonized endpoints, curated databases, and transparent preprocessing pipelines are prerequisites for credible AI models and reproducible systems pharmacology. Second, multi-target design should be intentional rather than incidental; multi-objective optimization, uncertainty-aware prioritization, and explicit off-target risk assessment are necessary to balance efficacy, safety, and developability. Third, the most impactful studies will tightly integrate computation and experimentation, translating omics-derived hypotheses into a small number of high-confidence targets for directionality-aware testing and subjecting AI-prioritized compounds to prospective evaluation, including early ADMET screening. Finally, openness should be the norm: shared code, datasets, and validation protocols will facilitate independent replication and rapid methodological iteration.

Together, the six articles create a cohesive impact by supporting a realistic message: finding natural medicines to treat metabolic disorders is most effective when computational prioritization, inference of systems-level mechanisms, and pharmacological validation are integrated into a single auditable process. The Volume II Research Topic is expected to help increase reproducible, mechanism-based pipelines and accelerate the development of promising natural products into plausible therapeutic candidates.
